# Editorial Notes

**Published:** 1903-03

**Authors:** 


### Editorial Notes.

**University College Colston Society.**

THE question of our National Education is just now, fortunately for our country, an uppermost topic in the mind of every thinking man; hence a special interest attaches to the Fourth Annual Meeting of this Society, which has a twofold object—the wider support of the University College Bristol, and the furthering of the scheme for a Western University in Bristol.

The President for the year was the LORD BISHOP OF BRISTOL, himself a weighty authority on Educational matters. In the course of his speech he said that they talked about the nation falling behind in commercial enterprise and success. He felt strongly that technical education should be more rightly termed technical teaching, which was exceedingly good for the training of hands. It was not, however, hands that would dominate commerce: it was brains, and it was brains that they had to develop. There was no question at all that in commerce, in warfare, and in all other departments, brains were now ruling the world; and more and more would this be so. Unless they could bring to the services of the country, in every walk in life, a larger proportion of brains than up to this time had been done, they were bound to go behind other nations which recognise that brains rule, and would continue to rule, the world.

Mr. J. W. ARROWSMITH, the indefatigable honorary secretary, to whose initiation the Society owed its birth, was happily enabled to announce several additional special donations to University College, which, with sums promised subsequent to the dinner, provided the £3,000 necessary to secure the generous offers of £1,000 each from Sir W. H. Wills, Bart., and Sir Frederick Wills, Bart., M.P., to which we alluded in a previous issue. Moreover, the subscriptions to the Society for the year exceeded £400. All this shows that the Colston Society has proved a very practical medium for obtaining valuable additional support for the University College; and we may cordially congratulate the President, the Lord Bishop of Bristol, and Mr. J. W. Arrowsmith, on a very successful year's proceedings.

*List of Special Donations to University College.*

| | £ | s. | d. | | £ | s. | d. |
|---|---|---|---|---|---|---|---|
| Wills, Sir W. H., Bart.... | 1000 | 0 | 0 | Baker, Hiatt C., J.P. ... | 250 | 0 | 0 |
| Wills, Sir Frederick, Bart., M.P. ... ... ... | 1000 | 0 | 0 | Wills, G. A., J.P. ... ... | 100 | 0 | 0 |
| Fry, Francis J., J.P. ... | 500 | 0 | 0 | Robinson, Arthur ... ... | 50 | 0 | 0 |
| Fry, J. Storrs ... ... ... | 500 | 0 | 0 | Wills, H. H. ... .. ... | 50 | 0 | 0 |
| Fry, Right Hon. Lewis... | 250 | 0 | 0 | The High Sheriff of Bristol | 10 | 10 | 0 |
| Fry, Albert, J.P. ... ... | 250 | 0 | 0 | Swann, E. J., J.P. ... ... | 10 | 10 | 0 |
| Fry, A. M. ... .. ... | 250 | 0 | 0 | Fry, G. Falconer ... ... | 10 | 10 | 0 |
| Worsley, P. J., J.P. ... | 250 | 0 | 0 | Anonymous ... ... ... | 5 | 0 | 0 |
| Thomas, Harry E.... ... | 250 | 0 | 0 | Nash, Herbert ... ... | 5 | 0 | 0 |
| Arrowsmith, J. W., J.P. | 250 | 0 | 0 | Whittuck, A. E. ... ... | 5 | 0 | 0 |
| Robinson, Edward, J.P. | 250 | 0 | 0 | The Lord Bishop of Bristol | *3 | 10 | 0 |
| Shipley, A., J.P. ... ... | 250 | 0 | 0 | | £5500 | 0 | 0 |

* To make total £5500 : 0 : 0

The Right Hon. H. Hobhouse, M.P., strongly supported the movement for a local university. This was the moment when University College might hold out its hands to the adjoining counties. In the West of England Bristol had an unrivalled situation and an unrivalled opportunity. They had merchant princes still alive in the good old city, and he believed Mr. Arrowsmith's wish would be realised. A provincial university would be the university of the future, and Bristol would be known as the educational capital of the West of England.

Sir J. Crichton Browne, F.R.S., the guest of the evening, remarked that the University College had made itself responsible for a scheme of great magnitude, of far-reaching significance—a scheme for a university located in the city of Bristol. They were all familiar with the objections to the multiplication of universities; but these objections came a little too late in the day, and they were futile in view of the pressing educational wants of the hour. Bristol had long possessed breadth and culture greater than some other towns already endowed with universities. They wanted a group of national universities, which should collectively meet the higher educational needs of the whole country—a group of universities, each specially adapted to its environment, but all affording the amplest instruction, the finest culture, the best discipline of the day. And that group of English universities would be incomplete if a university of Bristol did not find a place in it. If Liverpool obtained the charter they asked for, then, without doubt, they would have modern universities at Liverpool, Leeds, Manchester, Durham, Birmingham, and Cardiff. A great educational upheaval was going on. An extension of their English university system was inevitable; and it was to be hoped that a university of Bristol would make its appearance. Their Merchant Venturers' Technical College was, he believed, unsurpassed in the liberality of its arrangements, in buildings, equipment, and teaching power. Their University College was excellent: it was fortunate in having had on its professionale a number of men of marked ability, and some who had advanced to European reputation.

**Bristol's Awakening:** This phrase is just now in the mouths of every Bristolian in connection with the proposed new railway. We are not now concerned with the arguments in favour of or against such a scheme; but one thing is certain, that no commercial awakening can be completely successful that does not recognise, as Scotland and Germany have long realised in a very practical manner and with corresponding commercial success, that universities for the people are essential factors in the development of brains. It is not Bristol alone that is so deeply concerned in the promotion of this University Scheme, but the whole West of England; and if some one or more individuals, who have it in their power to do so, would give a large sum sufficient to immediately ensure the successful application for a charter, it would undoubtedly do a great deal for the commercial prosperity, as well as the culture and enlightenment, of the West of England, and benefit all classes of the community.

**Ventilation of Sewers.** ATTENTION has recently been directed by the *Lancet* Special Sanitary Commission (January 17th, 1903) to the fact that Bristol has been a pioneer in developing the idea that it is not necessary to take any trouble whatever about ventilating sewers, for the favourable vital statistics of Bristol are obtained in spite of the statement that there is no ventilation of any kind for the sewers. It is admitted, however, that an air-tight sewer is an impossibility: it is true that there are no grids ventilating the sewers on the street levels; it is even true that there are no ventilating shafts constructed for the purpose; but it is not true that there is no ventilation of the sewers. Statistics do not supply any evidence that the unconscious ventilation prevalent at Bristol gives worse results than the conscious ventilation applied in other towns. But it is pointed out that "every town has not got a tidal river to occasion the pumping of air in and out of the sewers twice a day. Nor is every town built at the bottom and up the sides of a basin as is Bristol. Every town has not the same proportion of newly built self-cleansing sewers running down precipitous hills. Every town has not got the same proportion of soil-pipes prolonged over the roof of the houses ready to act as sewer ventilators as in Bristol; and, above all, every town is not constantly swept and purified by the pure sea winds from the Bristol Channel."

**Electro-Therapeutic Work in Medicine and Surgery.**

GREAT developments are being made in the adaptation of light and electricity to therapeutic purposes. In spite of the reasonable prejudice which exists against methods which have been so much be-quacked in the past, we are bound to admit that the recent adaptations of electricity are such that we cannot afford to leave them in the hands of the charlatan, and they are becoming most valuable in the treatment of conditions which are for the most part highly intractable to other methods. We desire to direct attention to an outline of recent electro-therapeutic work given by Dr. John Macintyre (*Glasgow M. J.*, lviii., 1902, 341); and the paper by Dr. A. J. Harrison (p. 23) gives some of the earlier results obtained by means of the excellent installation which the munificence of a Bristol citizen has placed at the disposal of the staff of the Bristol General Hospital. We hope to receive from time to time further reports of the good work now being carried on in these departments at both of our large hospitals.

**The Therapeutics of a Sea Voyage.**

Dr. Roxburgh directs our attention (page 1) to a topic which is getting to be a little out of date. Thirty years ago the sea voyage was everything in the treatment of chest diseases, but the high-altitude methods became popular, and the sea level correspondingly became unpopular. But now that altitude has been declared to be nothing, and abundance of fresh air everything, there is every reason why the sea voyage should be reinstated in its former position as one of the best methods of treatment of tubercular diseases. The deck of a ship in motion must be the ideal spot for a patient requiring air without fatigue; and therefore a succession of voyages should be excellent treatment for our consumptives, if it be adopted early and not as a dernier resort. Some comments on "long sea voyages for chest diseases" made by the writer in 1876 (*vide Brit. M. J.*, 1876, ii. 360) are still appropriate, and more especially as to the necessity for early diagnosis and treatment in order that the treatment by sea voyage may be successful.

**Winsley Sanatorium for Consumptives.**

The Committee of the Gloucester, Somerset, and Wilts branch of the National Association for the Prevention of Consumption hope to commence building the Sanatorium at Winsley immediately. It has been found desirable to somewhat modify the original scheme, and the new plans have been submitted to the Committee which provide for the erection of an administration block for sixty patients and a building to contain twenty beds. The blocks for the remaining forty beds, to complete the scheme, can be added when the necessary funds have been raised.

The donations promised and paid up to the present amount to £9,437, and £3,649 has been expended in the purchase of land, preparation of the site, surveyor's fees, legal charges, advertising, and secretarial expenses, etc., leaving a balance of

£5,788. As at least £6,500 is required for the erection of the administration block and accommodation for twenty patients, exclusive of furniture and fittings, a large additional sum is required immediately. There is no doubt that the Sanatorium is urgently needed, and we have good reason to expect that the philanthropic public will provide the requisite amount, which is really very small when one considers that the whole of three populous counties are directly interested in the work.

**Notification of Phthisis.**

Compulsory notification does not meet with general approval, and has generally been considered to be unnecessary and impracticable. A modified voluntary method has been adopted in many places, but can only be of partial application and of doubtful utility. A writer in the *Medical Review* (January, 1903, p. 2) thinks that "compulsory notification of every case of tuberculosis is unnecessary and undesirable. But notification of all cases of 'tuberculosis with discharge' would be beneficial if isolation were enforced and the necessary accommodation provided. Whether notification of tuberculosis is enforced or not every room in which a tuberculous patient has died should be disinfected. In Vienna this has long been done by the authorities from information derived from death certificates, without the aid of notification. If any technical difficulties exist in England in employing death certificates for this purpose it would be easy to make it incumbent on the practitioner to notify to the sanitary authority every case of death from tuberculosis."

**Notification in Bristol.**

The following extracts from the Report to the Health Committee on the Notification of Phthisis, from Dr. D. S. Davies, the Medical Officer of Health of the City of Bristol, are of general as well as of local interest.

"Your Committee will remember that in April, 1899, I pointed out that initial measures towards notification had been attempted by the distribution of leaflets since 1891, prescribing the necessary precautions to be observed in regard to consumption, and by offering disinfection in houses where a consumptive person has resided, or after death. Following the suggestion of that Report, your Committee authorised the taking of samples of incoming milk from the surrounding districts for tubercle, and this was duly carried out for several months, fortunately with very encouraging results as to the apparent absence of tubercle from the great majority of such supplies. It was also resolved to frame all milk and meat contracts for the city hospitals on lines protective against tubercle. The Report also advised the provision of public slaughter-houses and the adoption of a voluntary system of notification of phthisis, but these suggestions were not at that time adopted.

Several districts have since made experiment in such voluntary notification, and with their experience to guide us, it is, I consider, expedient to consider definitely the adoption of this procedure in Bristol, as it is generally found that, with the necessary medical organisation, considerable benefit may be expected to result to the health of the district.

The plan found most effective in introducing voluntary notification is:—

1st. To circulate to every house in the city a copy of my leaflet of simple precautions to be observed in regard to consumptive patients, and in regard to houses inhabited or vacated by consumptive patients or in which consumptive patients may have died.

2nd. To circulate amongst publicans, owners and occupiers of workshops and workplaces, on public hoardings and in other public places, notices and handbills calling attention to simple precautions, and especially as to the danger of spitting.

3rd. After an interval of six months, again to distribute leaflets to every house in the city.

4th. To then invite notification of phthisis (consumption of the lungs only) from the district Medical Officer of the Guardians, and from the Medical Officer of dispensaries.

5th. At the same time to make provision for the examination of samples of phthisical (consumptive) expectoration free for medical men.

6th. To then finally initiate a general system of voluntary notification, together with the necessary administrative complement, medical, clerical, inspectorial and cleansing, for the due execution of the detailed work thus demanded. These details I am prepared to advise upon as they become necessary.

The question of sanatoria hinges very largely upon notification, from which it is possible not only to learn the extent of sanatorium accommodation called for, but also to get early information of cases, so as to control them in the stages when preventive treatment is most likely to succeed."

THE appointment of Mr. Leopold Statton as Chief Clerk to the department of the Medical Officer of Health for Bristol is an incident in the history of the Bristol Medical Library which calls for notice in these pages. From 1893, when the Library, as we know it, was formed, Mr. Statton as its clerk has been a real help to all its frequenters. He came to it equipped with an extensive knowledge of library work, acquired at our Museum and Library during many years, and he soon added to that knowledge a thorough bibliographical familiarity with our medical books and the special needs of the Library. His readiness to place his information at the service of anyone seeking his assistance made it a real pleasure to turn to him in any bibliographical difficulty. We are sorry to lose him, but wish him all success and happiness in his new work.

Mr. Fred. Farr, who worked with Mr. Statton for several years as assistant, has been appointed as the Librarian.

**Gordon Memorial College, Khartoum.**

DR. ANDREW BALFOUR, of Edinburgh, has been appointed Director of the Chemical and Bacteriological Research Laboratories of the above College. These laboratories are designed "to promote technical education and the study, bacteriologically and physiologically, of tropical disorders, especially the infective diseases of both man and beast peculiar to the Soudan, and to render assistance to the officers of health and to the staff of the civil and military hospitals; to aid criminal investigations in poisoning cases (which are frequent in the Soudan) by the detection and experimental determination of toxic agents, particularly the obscure potent substances employed by the natives; to carry out such tests in connection with water, food stuffs, and health and sanitary matters as may be found desirable; and to undertake the testing and assaying of agricultural, mineral, and other substances of practical interest in the industrial development of the Soudan." It will be remembered that the foundation of these laboratories was due to the scientific enthusiasm and generosity of Mr. Henry S. Wellcome. We believe that, with Dr. Balfour as director, the work done in these laboratories will do much for the development of the Soudan and for the general benefit of mankind.

